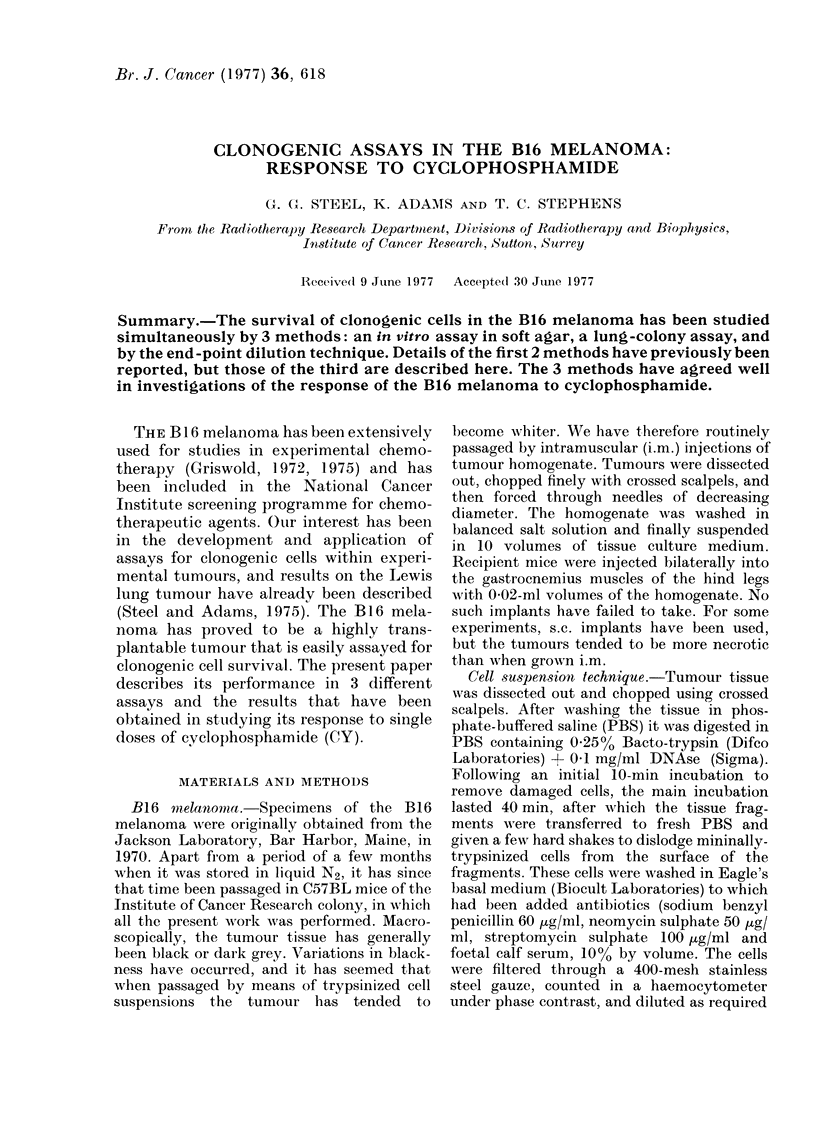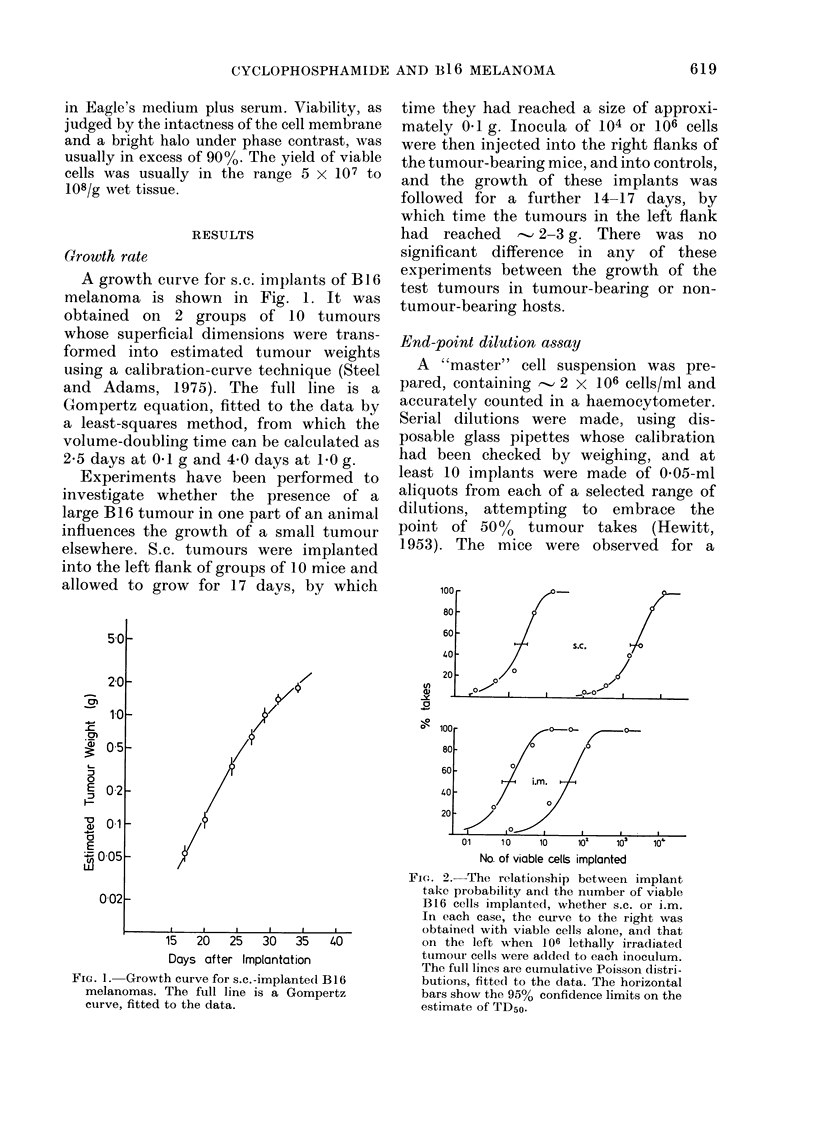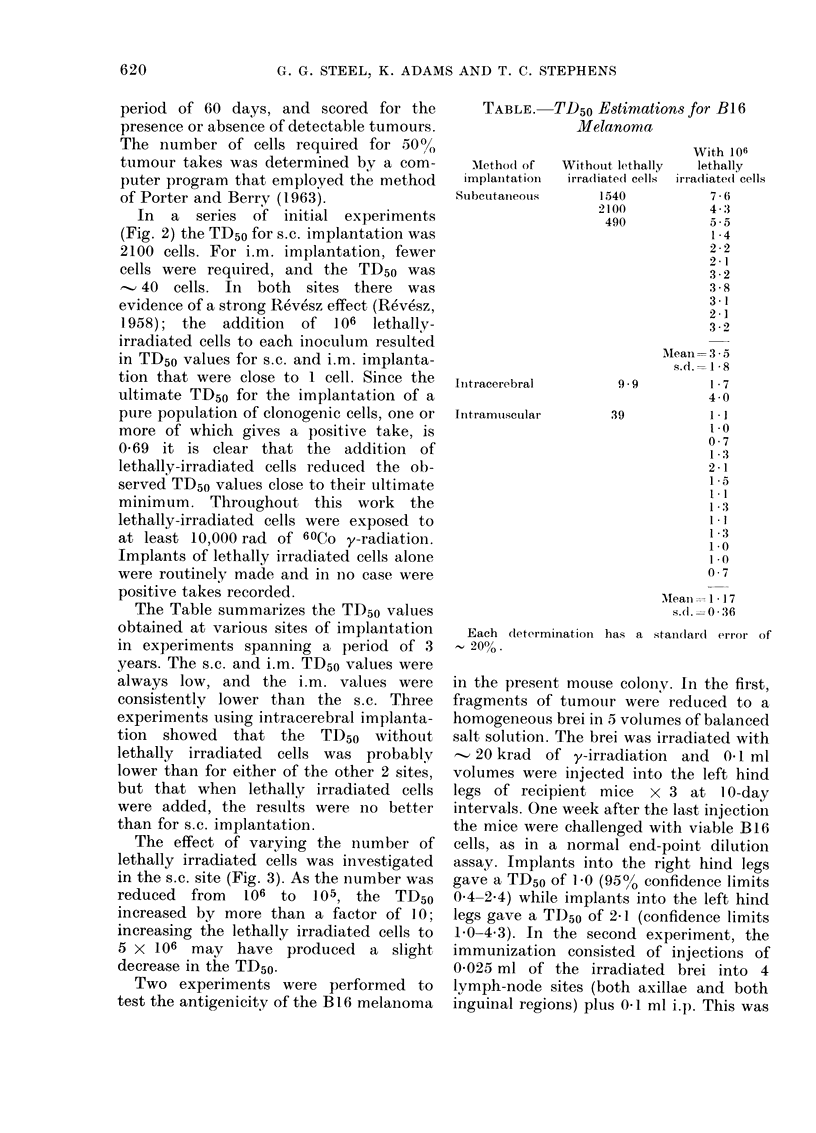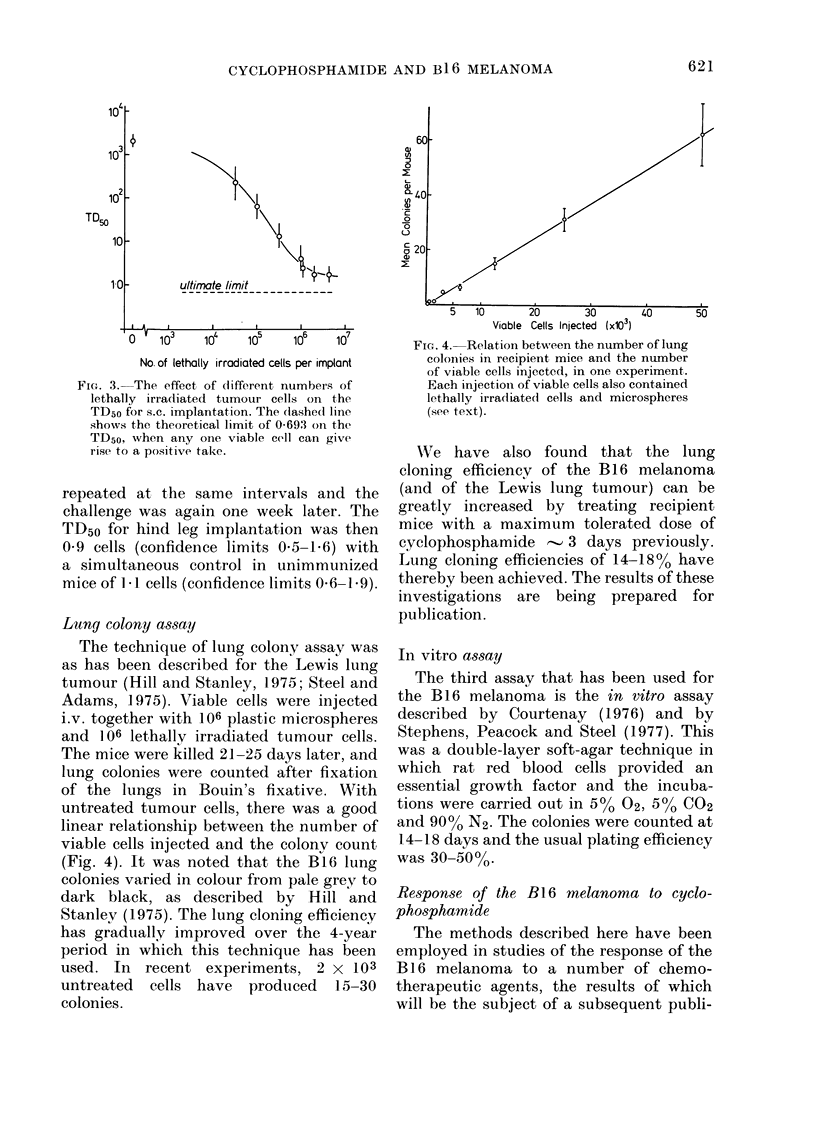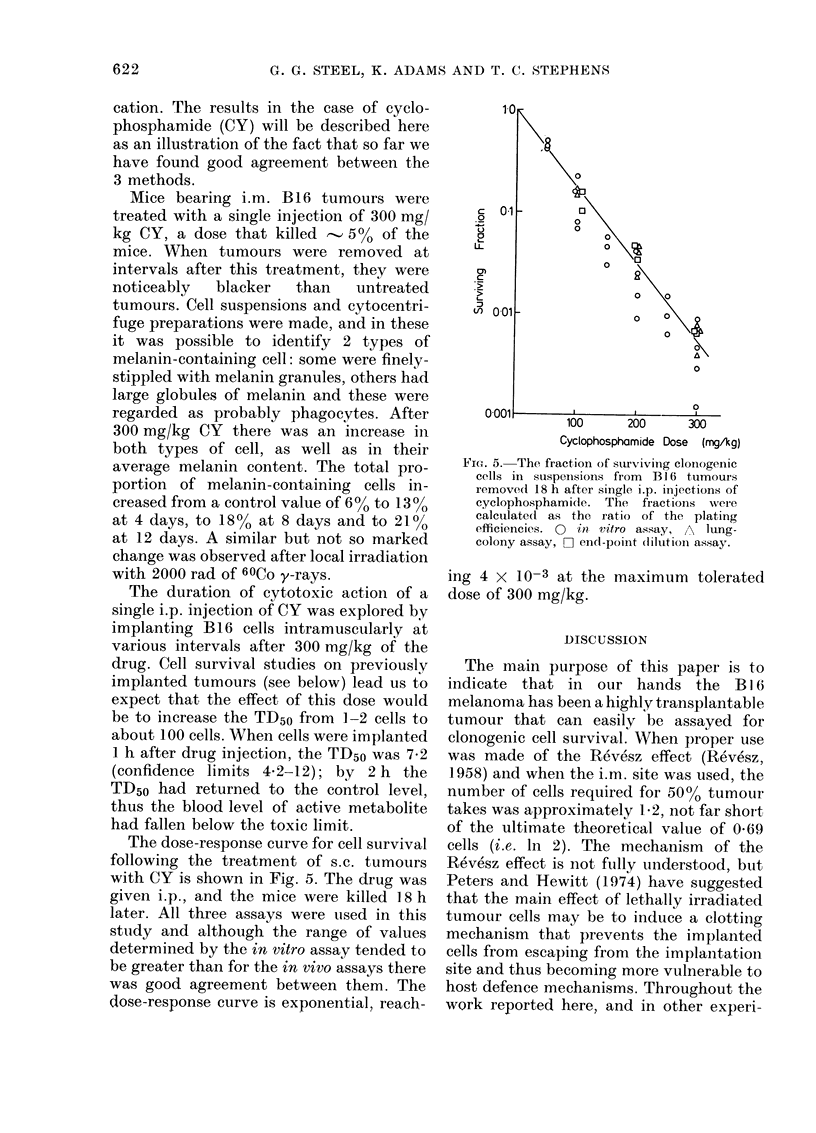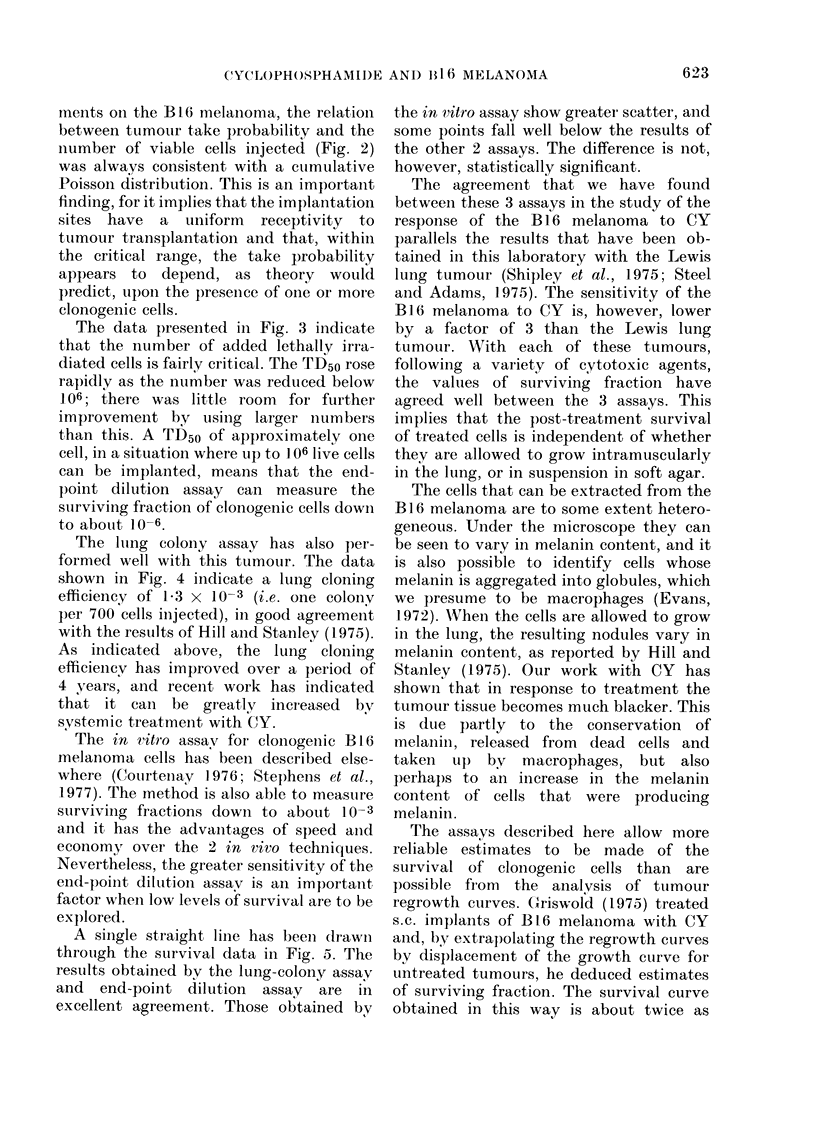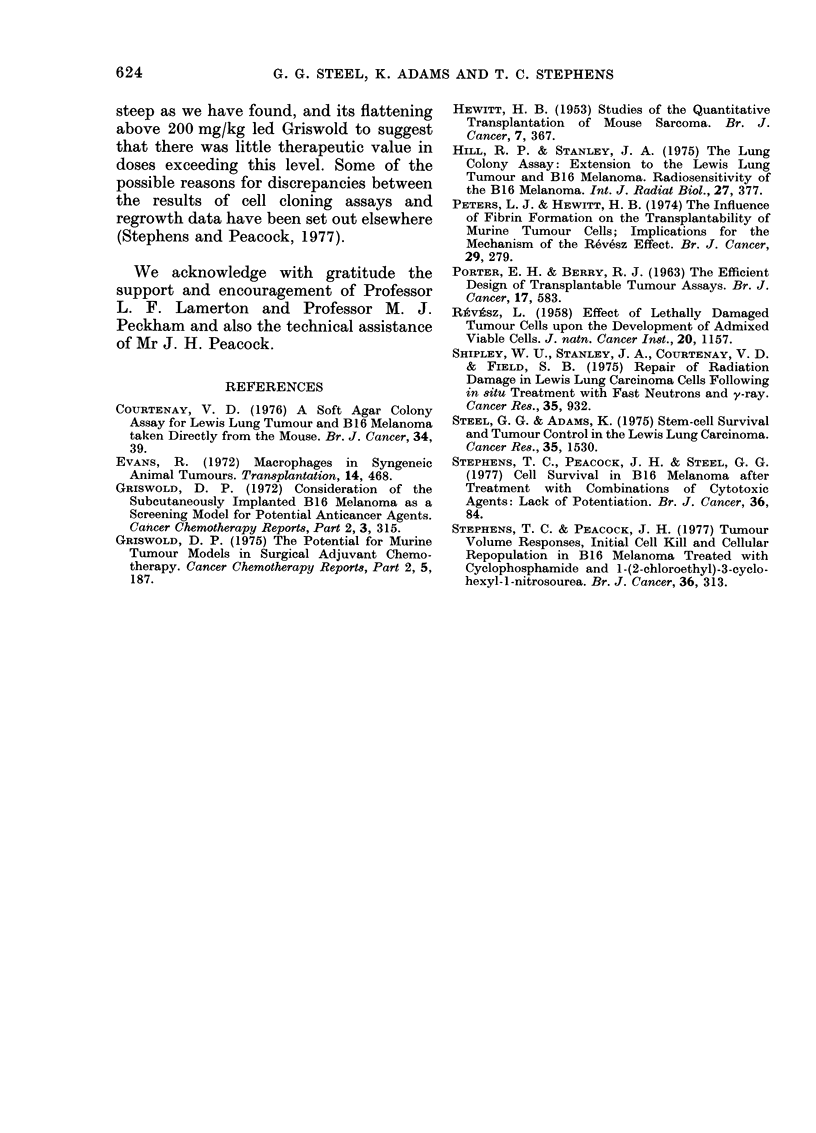# Clonogenic assays in the B16 melanoma: response to cyclophosphamide.

**DOI:** 10.1038/bjc.1977.239

**Published:** 1977-11

**Authors:** G. G. Steel, K. Adams, T. C. Stephens

## Abstract

The survival of clonogenic cells in the B16 melanoma has been studied simultaneously by 3 methods: an in vitro assay in soft agar, a lung-colony assay, and the end-point dilution technique. Details of the first 2 methods have previously been reported, but those of the third are described here. The 3 methods have agreed well in investigations of the response of the B16 melanoma to cyclophosphamide.


*Br. J. Cancer* (1977) **36**, 618

### CLONOGENIC ASSAYS IN THE B16 MELANOMA: RESPONSE TO CYCLOPHOSPHAMIDE

G. G. STEEL, K. ADAMS AND T. C. STEPHENS

*From the Radiotherapy Research Department, Divisions of Radiotherapy and Biophysics, Institute of Cancer Research, Sutton, Surrey*

Received 9 June 1977 Accepted 30 June 1977

**Summary.—The survival of clonogenic cells in the B16 melanoma has been studied simultaneously by 3 methods: an *in vitro* assay in soft agar, a lung-colony assay, and by the end-point dilution technique. Details of the first 2 methods have previously been reported, but those of the third are described here. The 3 methods have agreed well in investigations of the response of the B16 melanoma to cyclophosphamide.**

THE B16 melanoma has been extensively used for studies in experimental chemotherapy (Griswold, 1972, 1975) and has been included in the National Cancer Institute screening programme for chemotherapeutic agents. Our interest has been in the development and application of assays for clonogenic cells within experimental tumours, and results on the Lewis lung tumour have already been described (Steel and Adams, 1975). The B16 melanoma has proved to be a highly transplantable tumour that is easily assayed for clonogenic cell survival. The present paper describes its performance in 3 different assays and the results that have been obtained in studying its response to single doses of cyclophosphamide (CY).

### MATERIALS AND METHODS

*B*16 *melanoma*.—Specimens of the B16 melanoma were originally obtained from the Jackson Laboratory, Bar Harbor, Maine, in 1970. Apart from a period of a few months when it was stored in liquid $N_2$, it has since that time been passaged in C57BL mice of the Institute of Cancer Research colony, in which all the present work was performed. Macroscopically, the tumour tissue has generally been black or dark grey. Variations in blackness have occurred, and it has seemed that when passaged by means of trypsinized cell suspensions the tumour has tended to become whiter. We have therefore routinely passaged by intramuscular (i.m.) injections of tumour homogenate. Tumours were dissected out, chopped finely with crossed scalpels, and then forced through needles of decreasing diameter. The homogenate was washed in balanced salt solution and finally suspended in 10 volumes of tissue culture medium. Recipient mice were injected bilaterally into the gastrocnemius muscles of the hind legs with 0·02-ml volumes of the homogenate. No such implants have failed to take. For some experiments, s.c. implants have been used, but the tumours tended to be more necrotic than when grown i.m.

*Cell suspension technique*.—Tumour tissue was dissected out and chopped using crossed scalpels. After washing the tissue in phosphate-buffered saline (PBS) it was digested in PBS containing 0·25% Bacto-trypsin (Difco Laboratories) + 0·1 mg/ml DNAse (Sigma). Following an initial 10-min incubation to remove damaged cells, the main incubation lasted 40 min, after which the tissue fragments were transferred to fresh PBS and given a few hard shakes to dislodge mininally-trypsinized cells from the surface of the fragments. These cells were washed in Eagle's basal medium (Biocult Laboratories) to which had been added antibiotics (sodium benzyl penicillin 60 $\mu$g/ml, neomycin sulphate 50 $\mu$g/ml, streptomycin sulphate 100 $\mu$g/ml and foetal calf serum, 10% by volume. The cells were filtered through a 400-mesh stainless steel gauze, counted in a haemocytometer under phase contrast, and diluted as required in Eagle's medium plus serum. Viability, as judged by the intactness of the cell membrane and a bright halo under phase contrast, was usually in excess of 90%. The yield of viable cells was usually in the range $5 \times 10^7$ to $10^8$/g wet tissue.

### RESULTS

### *Growth rate*

A growth curve for s.c. implants of B16 melanoma is shown in Fig. 1. It was obtained on 2 groups of 10 tumours whose superficial dimensions were transformed into estimated tumour weights using a calibration-curve technique (Steel and Adams, 1975). The full line is a Gompertz equation, fitted to the data by a least-squares method, from which the volume-doubling time can be calculated as 2·5 days at 0·1 g and 4·0 days at 1·0 g.

Experiments have been performed to investigate whether the presence of a large B16 tumour in one part of an animal influences the growth of a small tumour elsewhere. S.c. tumours were implanted into the left flank of groups of 10 mice and allowed to grow for 17 days, by which time they had reached a size of approximately 0·1 g. Inocula of $10^4$ or $10^6$ cells were then injected into the right flanks of the tumour-bearing mice, and into controls, and the growth of these implants was followed for a further 14–17 days, by which time the tumours in the left flank had reached ~ 2–3 g. There was no significant difference in any of these experiments between the growth of the test tumours in tumour-bearing or non-tumour-bearing hosts.

### *End-point dilution assay*

A "master" cell suspension was prepared, containing ~ $2 \times 10^6$ cells/ml and accurately counted in a haemocytometer. Serial dilutions were made, using disposable glass pipettes whose calibration had been checked by weighing, and at least 10 implants were made of 0·05-ml aliquots from each of a selected range of dilutions, attempting to embrace the point of 50% tumour takes (Hewitt, 1953). The mice were observed for a

FIG. 1.—Growth curve for s.c.-implanted B16 melanomas. The full line is a Gompertz curve, fitted to the data.

FIG. 2.—The relationship between implant take probability and the number of viable B16 cells implanted, whether s.c. or i.m. In each case, the curve to the right was obtained with viable cells alone, and that on the left when $10^6$ lethally irradiated tumour cells were added to each inoculum. The full lines are cumulative Poisson distributions, fitted to the data. The horizontal bars show the 95% confidence limits on the estimate of $TD_{50}$.

period of 60 days, and scored for the presence or absence of detectable tumours. The number of cells required for 50% tumour takes was determined by a computer program that employed the method of Porter and Berry (1963).

In a series of initial experiments (Fig. 2) the $TD_{50}$ for s.c. implantation was 2100 cells. For i.m. implantation, fewer cells were required, and the $TD_{50}$ was $\sim 40$ cells. In both sites there was evidence of a strong Révész effect (Révész, 1958); the addition of $10^6$ lethally-irradiated cells to each inoculum resulted in $TD_{50}$ values for s.c. and i.m. implantation that were close to 1 cell. Since the ultimate $TD_{50}$ for the implantation of a pure population of clonogenic cells, one or more of which gives a positive take, is 0·69 it is clear that the addition of lethally-irradiated cells reduced the observed $TD_{50}$ values close to their ultimate minimum. Throughout this work the lethally-irradiated cells were exposed to at least 10,000 rad of $^{60}Co$ $\gamma$-radiation. Implants of lethally irradiated cells alone were routinely made and in no case were positive takes recorded.

The Table summarizes the $TD_{50}$ values obtained at various sites of implantation in experiments spanning a period of 3 years. The s.c. and i.m. $TD_{50}$ values were always low, and the i.m. values were consistently lower than the s.c. Three experiments using intracerebral implantation showed that the $TD_{50}$ without lethally irradiated cells was probably lower than for either of the other 2 sites, but that when lethally irradiated cells were added, the results were no better than for s.c. implantation.

The effect of varying the number of lethally irradiated cells was investigated in the s.c. site (Fig. 3). As the number was reduced from $10^6$ to $10^5$, the $TD_{50}$ increased by more than a factor of 10; increasing the lethally irradiated cells to $5 \times 10^6$ may have produced a slight decrease in the $TD_{50}$.

Two experiments were performed to test the antigenicity of the B16 melanoma

TABLE.—*$TD_{50}$ Estimations for B16 Melanoma*

| Method of implantation | Without lethally irradiated cells | With $10^6$ lethally irradiated cells |
|---|---|---|
| Subcutaneous | 1540 | 7·6 |
| | 2100 | 4·3 |
| | 490 | 5·5 |
| | | 1·4 |
| | | 2·2 |
| | | 2·1 |
| | | 3·2 |
| | | 3·8 |
| | | 3·1 |
| | | 2·1 |
| | | 3·2 |
| | | Mean = 3·5 |
| | | s.d. = 1·8 |
| Intracerebral | 9·9 | 1·7 |
| | | 4·0 |
| Intramuscular | 39 | 1·1 |
| | | 1·0 |
| | | 0·7 |
| | | 1·3 |
| | | 2·1 |
| | | 1·5 |
| | | 1·1 |
| | | 1·3 |
| | | 1·1 |
| | | 1·3 |
| | | 1·0 |
| | | 1·0 |
| | | 0·7 |
| | | Mean = 1·17 |
| | | s.d. = 0·36 |

Each determination has a standard error of $\sim 20\%$.

in the present mouse colony. In the first, fragments of tumour were reduced to a homogeneous brei in 5 volumes of balanced salt solution. The brei was irradiated with $\sim 20$ krad of $\gamma$-irradiation and 0·1 ml volumes were injected into the left hind legs of recipient mice $\times$ 3 at 10-day intervals. One week after the last injection the mice were challenged with viable B16 cells, as in a normal end-point dilution assay. Implants into the right hind legs gave a $TD_{50}$ of 1·0 (95% confidence limits 0·4–2·4) while implants into the left hind legs gave a $TD_{50}$ of 2·1 (confidence limits 1·0–4·3). In the second experiment, the immunization consisted of injections of 0·025 ml of the irradiated brei into 4 lymph-node sites (both axillae and both inguinal regions) plus 0·1 ml i.p. This was

FIG. 3.—The effect of different numbers of lethally irradiated tumour cells on the $TD_{50}$ for s.c. implantation. The dashed line shows the theoretical limit of 0·693 on the $TD_{50}$, when any one viable cell can give rise to a positive take.

FIG. 4.—Relation between the number of lung colonies in recipient mice and the number of viable cells injected, in one experiment. Each injection of viable cells also contained lethally irradiated cells and microspheres (see text).

repeated at the same intervals and the challenge was again one week later. The $TD_{50}$ for hind leg implantation was then 0·9 cells (confidence limits 0·5–1·6) with a simultaneous control in unimmunized mice of 1·1 cells (confidence limits 0·6–1·9).

### *Lung colony assay*

The technique of lung colony assay was as has been described for the Lewis lung tumour (Hill and Stanley, 1975; Steel and Adams, 1975). Viable cells were injected i.v. together with $10^6$ plastic microspheres and $10^6$ lethally irradiated tumour cells. The mice were killed 21–25 days later, and lung colonies were counted after fixation of the lungs in Bouin's fixative. With untreated tumour cells, there was a good linear relationship between the number of viable cells injected and the colony count (Fig. 4). It was noted that the B16 lung colonies varied in colour from pale grey to dark black, as described by Hill and Stanley (1975). The lung cloning efficiency has gradually improved over the 4-year period in which this technique has been used. In recent experiments, $2 \times 10^3$ untreated cells have produced 15–30 colonies.

We have also found that the lung cloning efficiency of the B16 melanoma (and of the Lewis lung tumour) can be greatly increased by treating recipient mice with a maximum tolerated dose of cyclophosphamide $\sim$ 3 days previously. Lung cloning efficiencies of 14–18% have thereby been achieved. The results of these investigations are being prepared for publication.

### In vitro *assay*

The third assay that has been used for the B16 melanoma is the *in vitro* assay described by Courtenay (1976) and by Stephens, Peacock and Steel (1977). This was a double-layer soft-agar technique in which rat red blood cells provided an essential growth factor and the incubations were carried out in 5% $O_2$, 5% $CO_2$ and 90% $N_2$. The colonies were counted at 14–18 days and the usual plating efficiency was 30–50%.

### *Response of the B16 melanoma to cyclophosphamide*

The methods described here have been employed in studies of the response of the B16 melanoma to a number of chemotherapeutic agents, the results of which will be the subject of a subsequent publication. The results in the case of cyclophosphamide (CY) will be described here as an illustration of the fact that so far we have found good agreement between the 3 methods.

Mice bearing i.m. B16 tumours were treated with a single injection of 300 mg/kg CY, a dose that killed ~5% of the mice. When tumours were removed at intervals after this treatment, they were noticeably blacker than untreated tumours. Cell suspensions and cytocentrifuge preparations were made, and in these it was possible to identify 2 types of melanin-containing cell: some were finely-stippled with melanin granules, others had large globules of melanin and these were regarded as probably phagocytes. After 300 mg/kg CY there was an increase in both types of cell, as well as in their average melanin content. The total proportion of melanin-containing cells increased from a control value of 6% to 13% at 4 days, to 18% at 8 days and to 21% at 12 days. A similar but not so marked change was observed after local irradiation with 2000 rad of $^{60}Co$ $\gamma$-rays.

The duration of cytotoxic action of a single i.p. injection of CY was explored by implanting B16 cells intramuscularly at various intervals after 300 mg/kg of the drug. Cell survival studies on previously implanted tumours (see below) lead us to expect that the effect of this dose would be to increase the $TD_{50}$ from 1–2 cells to about 100 cells. When cells were implanted 1 h after drug injection, the $TD_{50}$ was 7·2 (confidence limits 4·2–12); by 2 h the $TD_{50}$ had returned to the control level, thus the blood level of active metabolite had fallen below the toxic limit.

The dose-response curve for cell survival following the treatment of s.c. tumours with CY is shown in Fig. 5. The drug was given i.p., and the mice were killed 18 h later. All three assays were used in this study and although the range of values determined by the *in vitro* assay tended to be greater than for the *in vivo* assays there was good agreement between them. The dose-response curve is exponential, reaching $4 \times 10^{-3}$ at the maximum tolerated dose of 300 mg/kg.

FIG. 5.—The fraction of surviving clonogenic cells in suspensions from B16 tumours removed 18 h after single i.p. injections of cyclophosphamide. The fractions were calculated as the ratio of the plating efficiencies. ○ *in vitro* assay, △ lung-colony assay, □ end-point dilution assay.

### DISCUSSION

The main purpose of this paper is to indicate that in our hands the B16 melanoma has been a highly transplantable tumour that can easily be assayed for clonogenic cell survival. When proper use was made of the Révész effect (Révész, 1958) and when the i.m. site was used, the number of cells required for 50% tumour takes was approximately 1·2, not far short of the ultimate theoretical value of 0·69 cells (*i.e.* ln 2). The mechanism of the Révész effect is not fully understood, but Peters and Hewitt (1974) have suggested that the main effect of lethally irradiated tumour cells may be to induce a clotting mechanism that prevents the implanted cells from escaping from the implantation site and thus becoming more vulnerable to host defence mechanisms. Throughout the work reported here, and in other experiments on the B16 melanoma, the relation between tumour take probability and the number of viable cells injected (Fig. 2) was always consistent with a cumulative Poisson distribution. This is an important finding, for it implies that the implantation sites have a uniform receptivity to tumour transplantation and that, within the critical range, the take probability appears to depend, as theory would predict, upon the presence of one or more clonogenic cells.

The data presented in Fig. 3 indicate that the number of added lethally irradiated cells is fairly critical. The $TD_{50}$ rose rapidly as the number was reduced below $10^6$; there was little room for further improvement by using larger numbers than this. A $TD_{50}$ of approximately one cell, in a situation where up to $10^6$ live cells can be implanted, means that the end-point dilution assay can measure the surviving fraction of clonogenic cells down to about $10^{-6}$.

The lung colony assay has also performed well with this tumour. The data shown in Fig. 4 indicate a lung cloning efficiency of $1{\cdot}3 \times 10^{-3}$ (*i.e.* one colony per 700 cells injected), in good agreement with the results of Hill and Stanley (1975). As indicated above, the lung cloning efficiency has improved over a period of 4 years, and recent work has indicated that it can be greatly increased by systemic treatment with CY.

The *in vitro* assay for clonogenic B16 melanoma cells has been described elsewhere (Courtenay 1976; Stephens *et al.*, 1977). The method is also able to measure surviving fractions down to about $10^{-3}$ and it has the advantages of speed and economy over the 2 *in vivo* techniques. Nevertheless, the greater sensitivity of the end-point dilution assay is an important factor when low levels of survival are to be explored.

A single straight line has been drawn through the survival data in Fig. 5. The results obtained by the lung-colony assay and end-point dilution assay are in excellent agreement. Those obtained by the *in vitro* assay show greater scatter, and some points fall well below the results of the other 2 assays. The difference is not, however, statistically significant.

The agreement that we have found between these 3 assays in the study of the response of the B16 melanoma to CY parallels the results that have been obtained in this laboratory with the Lewis lung tumour (Shipley *et al.*, 1975; Steel and Adams, 1975). The sensitivity of the B16 melanoma to CY is, however, lower by a factor of 3 than the Lewis lung tumour. With each of these tumours, following a variety of cytotoxic agents, the values of surviving fraction have agreed well between the 3 assays. This implies that the post-treatment survival of treated cells is independent of whether they are allowed to grow intramuscularly in the lung, or in suspension in soft agar.

The cells that can be extracted from the B16 melanoma are to some extent heterogeneous. Under the microscope they can be seen to vary in melanin content, and it is also possible to identify cells whose melanin is aggregated into globules, which we presume to be macrophages (Evans, 1972). When the cells are allowed to grow in the lung, the resulting nodules vary in melanin content, as reported by Hill and Stanley (1975). Our work with CY has shown that in response to treatment the tumour tissue becomes much blacker. This is due partly to the conservation of melanin, released from dead cells and taken up by macrophages, but also perhaps to an increase in the melanin content of cells that were producing melanin.

The assays described here allow more reliable estimates to be made of the survival of clonogenic cells than are possible from the analysis of tumour regrowth curves. Griswold (1975) treated s.c. implants of B16 melanoma with CY and, by extrapolating the regrowth curves by displacement of the growth curve for untreated tumours, he deduced estimates of surviving fraction. The survival curve obtained in this way is about twice as steep as we have found, and its flattening above 200 mg/kg led Griswold to suggest that there was little therapeutic value in doses exceeding this level. Some of the possible reasons for discrepancies between the results of cell cloning assays and regrowth data have been set out elsewhere (Stephens and Peacock, 1977).

We acknowledge with gratitude the support and encouragement of Professor L. F. Lamerton and Professor M. J. Peckham and also the technical assistance of Mr J. H. Peacock.

### REFERENCES

COURTENAY, V. D. (1976) A Soft Agar Colony Assay for Lewis Lung Tumour and B16 Melanoma taken Directly from the Mouse. *Br. J. Cancer*, **34**, 39.

EVANS, R. (1972) Macrophages in Syngeneic Animal Tumours. *Transplantation*, **14**, 468.

GRISWOLD, D. P. (1972) Consideration of the Subcutaneously Implanted B16 Melanoma as a Screening Model for Potential Anticancer Agents. *Cancer Chemotherapy Reports, Part* 2, **3**, 315.

GRISWOLD, D. P. (1975) The Potential for Murine Tumour Models in Surgical Adjuvant Chemotherapy. *Cancer Chemotherapy Reports, Part* 2, **5**, 187.

HEWITT, H. B. (1953) Studies of the Quantitative Transplantation of Mouse Sarcoma. *Br. J. Cancer*, **7**, 367.

HILL, R. P. & STANLEY, J. A. (1975) The Lung Colony Assay: Extension to the Lewis Lung Tumour and B16 Melanoma. Radiosensitivity of the B16 Melanoma. *Int. J. Radiat Biol.*, **27**, 377.

PETERS, L. J. & HEWITT, H. B. (1974) The Influence of Fibrin Formation on the Transplantability of Murine Tumour Cells; Implications for the Mechanism of the Révész Effect. *Br. J. Cancer*, **29**, 279.

PORTER, E. H. & BERRY, R. J. (1963) The Efficient Design of Transplantable Tumour Assays. *Br. J. Cancer*, **17**, 583.

RÉVÉSZ, L. (1958) Effect of Lethally Damaged Tumour Cells upon the Development of Admixed Viable Cells. *J. natn. Cancer Inst.*, **20**, 1157.

SHIPLEY, W. U., STANLEY, J. A., COURTENAY, V. D. & FIELD, S. B. (1975) Repair of Radiation Damage in Lewis Lung Carcinoma Cells Following *in situ* Treatment with Fast Neutrons and $\gamma$-ray. *Cancer Res.*, **35**, 932.

STEEL, G. G. & ADAMS, K. (1975) Stem-cell Survival and Tumour Control in the Lewis Lung Carcinoma. *Cancer Res.*, **35**, 1530.

STEPHENS, T. C., PEACOCK, J. H. & STEEL, G. G. (1977) Cell Survival in B16 Melanoma after Treatment with Combinations of Cytotoxic Agents: Lack of Potentiation. *Br. J. Cancer*, **36**, 84.

STEPHENS, T. C. & PEACOCK, J. H. (1977) Tumour Volume Responses, Initial Cell Kill and Cellular Repopulation in B16 Melanoma Treated with Cyclophosphamide and 1-(2-chloroethyl)-3-cyclohexyl-1-nitrosourea. *Br. J. Cancer*, **36**, 313.